# Super-selective ASL and 4D ASL-based MR Angiography in a Patient with Moyamoya Disease: Case Report

**DOI:** 10.1007/s00062-020-00961-8

**Published:** 2020-09-25

**Authors:** Nico Sollmann, Hans Liebl, Christine Preibisch, Claus Zimmer, Michael Helle, Makoto Obara, Jan S. Kirschke, Stephan Kaczmarz

**Affiliations:** 1 Department of Diagnostic and Interventional Neuroradiology, Klinikum rechts der Isar, Technische Universität München, Ismaninger Str. 22, 81675 Munich, Germany; 2 TUM-Neuroimaging Center, Klinikum rechts der Isar, Technische Universität München, Munich, Germany; 3 Department of Radiology and Neuroradiology, University Hospital Schleswig-Holstein, Campus Kiel, Arnold-Heller-Str. 3, 24103 Kiel, Germany; 4 Philips Electronics Japan Healthcare, 13–37, Kohnan 2-chome, 108-8507 Tokyo, Japan

## Introduction

Moyamoya disease is a cerebrovascular disorder with progressive steno-occlusive affection of mainly the bilateral intracranial internal carotid artery (ICA) and its proximal branches [1, 2]. Moyamoya disease is predominantly observed in Japan, whereas the incidence in Europe is low and appears to amount to only one tenth of that observed in Japan [1, 3]. When moyamoya disease becomes clinically manifest, it entails symptoms due to brain ischemia (e.g., stroke) and/or due to compensatory mechanisms in response to ischemia (e.g., hemorrhage from fragile collateral vessels) [1].

Digital subtraction angiography (DSA) is the standard of reference for the diagnosis of moyamoya disease [1, 4]; however, computed tomography (CT) or, when rapidly available, magnetic resonance imaging (MRI) are the first-line modalities when a patient with neurological symptoms, potentially attributable to moyamoya disease, is referred for diagnosis. Time-of-flight (TOF) magnetic resonance angiography (MRA) is commonly used as the sequence of choice for evaluation of steno-occlusive vascular changes; however, it is static and cannot capture dynamic flow information, neither does it provide information about the potentially complex intracranial perfusion changes related to collateral vessels in moyamoya disease.

This article presents an imaging approach including super-selective arterial spin labeling (ASL) and super-selective 4D ASL-based MRA to non-invasively capture hemodynamic changes and time-resolved vessel architecture with a clinically applicable scan protocol in a patient with moyamoya disease.

## Case Presentation

### Clinical Symptoms

A 26-year-old German woman reported fluctuating intermittent hemihypesthesia of the right side of the body with acute onset. The patient did not show any focal neurological deficits during clinical examination. Her family anamnesis did not reveal any prior ischemic events, and migraine was the only known pre-existing condition.

### Magnetic Resonance Imaging

The patient was referred for MRI to screen for structural cerebral pathology, with the suspected diagnosis of ischemic stroke according to the neurological report at admission. Scanning was performed on a 3-Tesla system (Achieva dStream, software release R5.6; Philips Healthcare, Best, The Netherlands) using a 32-channel head coil and a custom patch (to facilitate super-selective imaging).

**Fig. 1:**
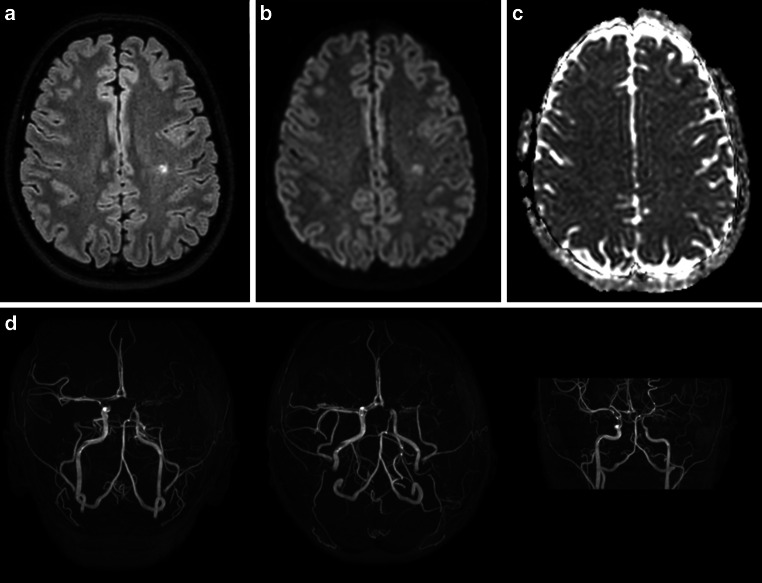
Fluid-attenuated inversion recovery (FLAIR; **a**) combined with diffusion-weighted imaging (**b**) and corresponding apparent diffusion coefficient map displayed in (**c**) revealed a subacute postischemic lesion in the left-hemispheric white matter. Time-of-flight (TOF) magnetic resonance angiography (MRA; **d**) showed multiple changes of arterial vascular calibers (with considerably reduced flow signals of the bilateral internal carotid artery [ICA] and its proximal branches)

The standard protocol included a 3D fluid-attenuated inversion recovery (FLAIR), TOF-MRA, diffusion-weighted imaging, and T2*-weighted sequence. The protocol revealed a FLAIR hyperintense (Fig. 1a) lesion in the left-hemispheric white matter (affecting the precentral and postcentral gyrus), which showed residual restricted diffusion (Fig. 1b,c), indicating subacute ischemia. In addition, TOF-MRA revealed multiple irregularities of arterial vascular calibers, most prominently captured by reduced flow signals of the bilateral ICA and its proximal branches (with the left-sided distal ICA showing discontinuous flow signal, indicative of pseudo-occlusive stenosis; Fig. 1d).

Due to these findings, non-selective pseudocontinuous ASL was applied according to the latest recommendations [5] (with manually set labeling plane based on phase contrast angiography, 1800 ms label duration, 2000 ms post-label delay, 2D gradient recalled echo [GRE] echo planar imaging [EPI] readout, TR/TE/alpha = 4500 ms/9.98 ms/90°, 3.3 × 3.6 × 6 mm^3^ spatial resolution, 0.6 mm gap, 16 slices, background suppression, EPI factor 29, scan time: 4 min 48 s). Furthermore, super-selective ASL (according to Helle et al. [6]; similar parameters as for pseudocontinuous ASL with 1800 ms post-label delay, scan time: 4 min 3 s) and 4D MRA based on super-selective ASL with contrast-enhanced timing-robust angiography (CENTRA) keyhole and view-sharing technique (4D-PACK according to Togao et al. [7]; 3D GRE readout, 7 time points at 100, 200, 500, 800, 1200, 1600 and 2000 ms, turbo factor 60, TR/TE/alpha = 4982 ms/1.7 ms/11°, 1 × 1.3 × 1.6 mm^3^ spatial resolution, scan time: 5 min 10 s) were separately applied for the left and right ICA. Labeling positions of super-selective acquisitions were successfully set fully automated based on TOF-MRA of the neck arteries (1.5 × 1.5 × 2 mm^3^ spatial resolution, acquired within 45 s with Compressed SENSE) by an additional software tool integrated in the scanner software.

**Fig. 2:**
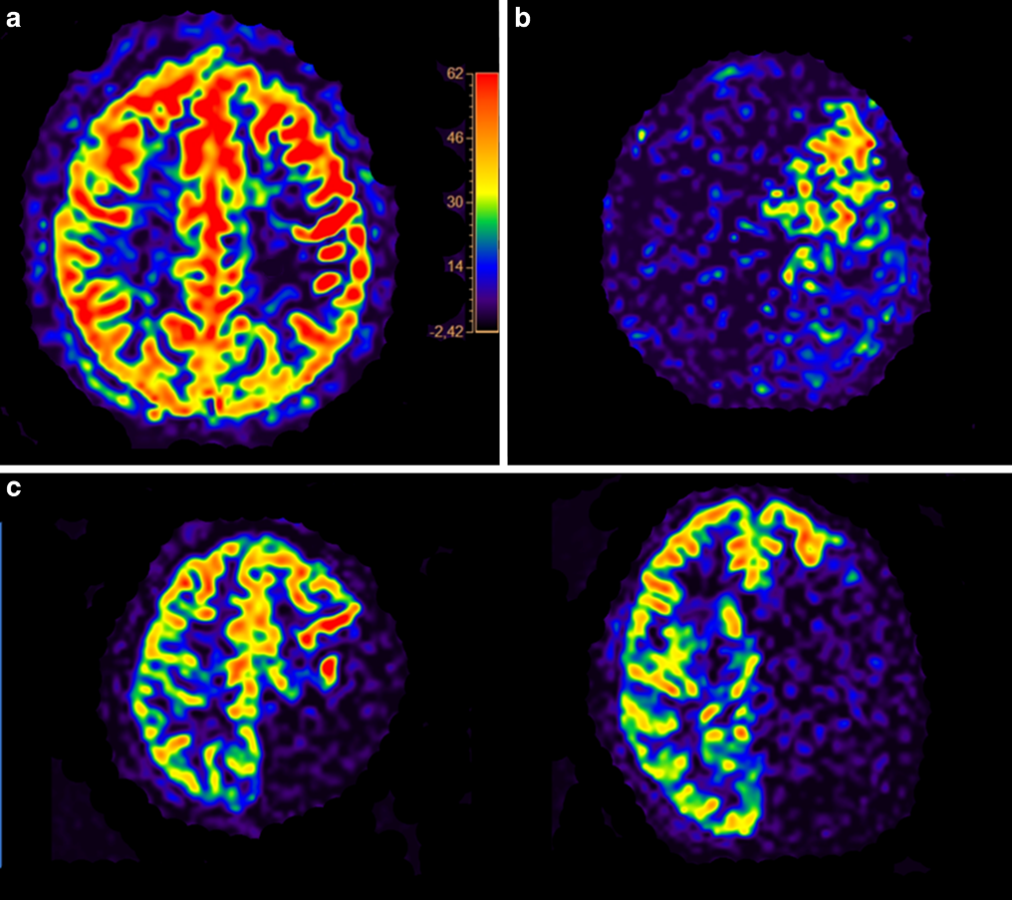
Pseudocontinuous arterial spin labeling (ASL) showed mostly normal cerebral blood flow (**a**; in ml/100 g/min), super-selective ASL showed reduced but detectable supply of the left middle cerebral artery territory by the left-sided internal carotid artery (ICA; **b**) and complete supply of the left anterior cerebral artery territory by the contralateral ICA (two representative axial slices; **c**)

**Fig. 3:**
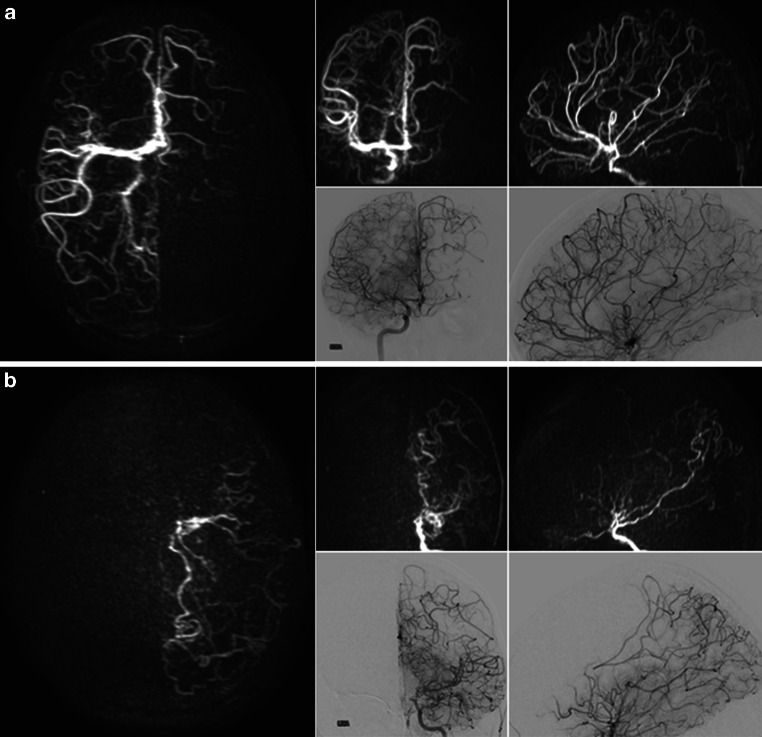
Super-selective 4D arterial spin labeling (ASL)-based magnetic resonance angiography (MRA; representative images with a delay of 2000 ms) and digital subtraction angiography (DSA) of the right-sided internal carotid artery (ICA; **a**) and left-sided ICA (**b**) showed aberrant interhemispheric supply patterns in time-resolved manner (takeover of the left anterior cerebral artery territory by branches of the contralateral hemisphere and prominent left-sided communicating posterior artery with retrograde filling of branches of the middle and anterior cerebral artery). Collaterals indicative of moyamoya disease were observed, primarily around the distal left-sided ICA and its proximal branches

Pseudocontinuous ASL revealed mostly normal cerebral blood flow globally (Fig. 2a), super-selective ASL was indicative of reduced but detectable supply of the left middle cerebral artery territory by the left-sided ICA and complete supply of the left anterior cerebral artery territory by the contralateral ICA (Fig. 2b,c). The super-selective 4D ASL-based MRA was able to capture the aberrant interhemispheric supply patterns in a time-resolved manner (takeover of the left anterior cerebral artery territory by branches of the contralateral hemisphere and prominent left-sided communicating posterior artery with retrograde filling of branches of the middle and anterior cerebral arteries; Fig. 3a, b). Furthermore, collaterals indicative of moyamoya disease were observed, most prominently originating around the distal left-sided ICA and its proximal branches (Fig. 3b). The super-selective 4D ASL-based MRA showed high signal intensity and enabled depiction of distal arterial branches (Fig. 3a, b).

**Fig. 4:**
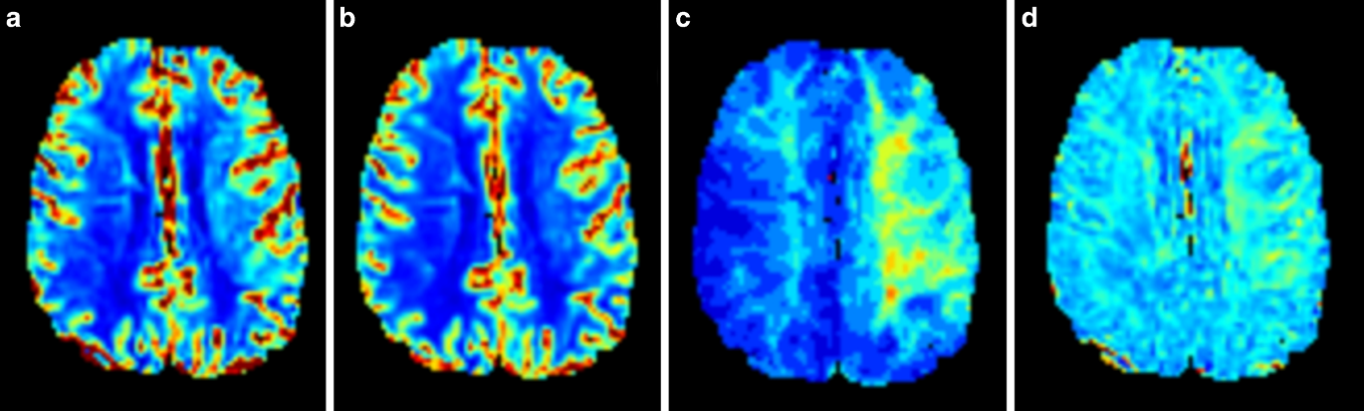
Dynamic susceptibility contrast (DSC) perfusion showing mostly normal to slighlty elevated cerebral blood volume (**a**) and cerebral blood flow (**b**), combined with an increased time to maximum (**c**) and mean transit time (**d**) for the left hemisphere

To rule out vasculitis, additional non-contrast-enhanced and contrast-enhanced T1-weighted black blood sequences were acquired, which did not show any vessel wall contrast enhancement. Furthermore, dynamic susceptibility contrast (DSC) perfusion (dynamic acquisition of 80 gradient-echo EPI volumes, TR/TE/alpha = 1483 ms/30 ms/60°, 26 slices, 2.0 × 2.0 × 3.5 mm^3^ spatial resolution, acquired within 2 min 3 s during injection of a weight-adjusted Gd-DOTA bolus) showed an increased time to maximum and mean transit time (Fig. 4a–d).

### Digital Subtraction Angiography

Two days after MRI acquisition, DSA of the brain-feeding arteries was performed (Azurion; Philips Healthcare, Best, The Netherlands). Coronal and lateral views were obtained after the injection of a bolus of iodinated contrast agent in the ICA and vertebral arteries of both sides. Very good visual concordance was found between images of the super-selective 4D ASL-based MRA and DSA (Fig. 3a, b).

## Discussion

In MRI of suspected cerebrovascular pathology, TOF-MRA is used for evaluation of steno-occlusive vascular changes during clinical routine. However, this sequence lacks reliability in visualizing slow blood flow or flow that is not directed in the feet-to-head direction, and it cannot provide dynamic flow information since it is a static technique. Hence, in disorders with changed brain perfusion patterns and development of collateral circulations, such as moyamoya disease, it may be of limited value.

In the presented patient case, a super-selective 4D ASL-based MRA was able to reveal time-resolved flow signals of the distal ICA that could predict the territorial perfusion and collateral vessels confirmed by DSA performed during the later course. Thus, prior to DSA, complete occlusion of the left-sided ICA could be ruled out as part of the primary diagnostic MRI examination, simultaneously displaying peripheral intracranial arterial branches as well as the formed collateral vessels characteristic of moyamoya disease. Of note, using super-selective ASL investigations of hemodynamic changes as well as for the depiction of the altered vessel architecture seem feasible as part of routine clinical MRI.

To the authors’ knowledge, the acquired 4D ASL-based MRA, referred to as 4D-PACK, has been used by only two preceding investigations [7, 8]; yet, in these two publications, no super-selective acquisitions were performed. One comparative study in Japanese patients with moyamoya disease investigated vessel visualization by the technique in relation to inflow-enhanced multi-phase angiography and DSA, pointing at significantly improved visualization of cerebral arteries and leptomeningeal collaterals for non-selective 4D-PACK [7]. High arterial signal in time-resolved manner can be obtained even at late time points because the sequence is based on pseudocontinuous ASL instead of pulsed ASL, and scanning is accelerated using the keyhole and view-sharing techniques [7, 8]. Thus, obtaining data for multiple post-labeling time points with clinically acceptable acquisition times becomes possible while achieving high spatial resolution with sufficient anatomical coverage and high vessel signal. However, the previous work did not apply the sequence in the acute setting, as done in the present investigation with very unclear blood supply patterns due to largely missing flow signal as depicted by TOF-MRA of the distal ICA, nor did it achieve a combination with super-selective ASL. In addition, the successfully applied novel automated labeling approach may particularly increase the feasibility in clinical routine, circumventing the need for time-consuming and sometimes error-prone manual labeling by specifically trained personal.

## Conclusion

The combination of super-selective ASL and super-selective 4D ASL-based MRA with automatic labeling presents a promising approach to evaluate hemodynamic changes and vessel architecture in a time-resolved manner with high resolution. The approach seems highly feasible for clinical routine MRI scan protocols. The super-selective 4D ASL-based MRA sequence allows immediate evaluation of selected arterial branches and collateral vessels in cerebrovascular diseases before DSA is performed, which is achieved without the need to apply a contrast agent.

## Abbreviations

ASL: Arterial spin labeling
CENTRA: Contrast-enhanced timing-robust angiography
CT: Computed tomography
DSA: Digital subtraction angiography
DSC: Dynamic susceptibility contrast
EPI: Echo planar imaging
FLAIR: Fluid-attenuated inversion recovery
GRE: Gradient recalled echo
ICA: Internal carotid artery
MRA: Magnetic resonance angiography
MRI: Magnetic resonance imaging
TOF: Time-of-flight
